# Effect of standardized training on the reliability of the Cochrane risk of bias assessment tool: a prospective study

**DOI:** 10.1186/s13643-017-0441-7

**Published:** 2017-03-03

**Authors:** Bruno R. da Costa, Brooke Beckett, Alison Diaz, Nina M. Resta, Bradley C. Johnston, Matthias Egger, Peter Jüni, Susan Armijo-Olivo

**Affiliations:** 1 Institute of Primary Health Care (BIHAM), University of Bern, Gesellschaftsstrasse 49, Bern, 3012 Switzerland; 2 Department of Physical Therapy, Florida International University, AHC3-430 11200 8 St, Miami, USA; 3 Department of Anesthesia and Pain Medicine, and Institute of Health Policy, Management and Evaluation, University of Toronto, Toronto, ON Canada; 4 Institute of Social and Preventive Medicine, University of Bern, Bern, Switzerland; 5 Applied Health Research Centre (AHRC), Li Ka Shing Knowledge Institute of St. Michael’s Hospital, Department of Medicine, University of Toronto, Toronto, Canada; 6 Faculty of Rehabilitation Medicine, Department of Physical Therapy, University of Alberta, Edmonton, AB Canada

## Abstract

**Background:**

The Cochrane risk of bias tool is commonly criticized for having a low reliability. We aimed to investigate whether training of raters, with objective and standardized instructions on how to assess risk of bias, can improve the reliability of the Cochrane risk of bias tool.

**Methods:**

In this pilot study, four raters inexperienced in risk of bias assessment were randomly allocated to minimal or intensive standardized training for risk of bias assessment of randomized trials of physical therapy treatments for patients with knee osteoarthritis pain. Two raters were experienced risk of bias assessors who served as reference. The primary outcome of our study was between-group reliability, defined as the agreement of the risk of bias assessments of inexperienced raters with the reference assessments of experienced raters. Consensus-based assessments were used for this purpose. The secondary outcome was within-group reliability, defined as the agreement of assessments within pairs of inexperienced raters. We calculated the chance-corrected weighted Kappa to quantify agreement within and between groups of raters for each of the domains of the risk of bias tool.

**Results:**

A total of 56 trials were included in our analysis. The Kappa for the agreement of inexperienced raters with reference across items of the risk of bias tool ranged from 0.10 to 0.81 for the minimal training group and from 0.41 to 0.90 for the standardized training group. The Kappa values for the agreement within pairs of inexperienced raters across the items of the risk of bias tool ranged from 0 to 0.38 for the minimal training group and from 0.93 to 1 for the standardized training group. Between-group differences in Kappa for the agreement of inexperienced raters with reference always favored the standardized training group and was most pronounced for incomplete outcome data (difference in Kappa 0.52, *p* < 0.001) and allocation concealment (difference in Kappa 0.30, *p* = 0.004).

**Conclusions:**

Intensive, standardized training on risk of bias assessment may significantly improve the reliability of the Cochrane risk of bias tool.

**Electronic supplementary material:**

The online version of this article (doi:10.1186/s13643-017-0441-7) contains supplementary material, which is available to authorized users.

## Background

Systematic reviews and meta-analyses of randomized clinical trials (RCTs) are central to evidence-based clinical decision-making [1, 2]. RCTs are the gold standard design for assessing the effectiveness of treatment interventions. Well-conducted RCTs may eliminate confounding, which allows decision-makers to infer that changes in the outcome of interest are causally linked with the experimental intervention. If results of RCTs included in a meta-analysis are biased, then the results of the meta-analysis will also be biased [3, 4]. Meta-analysis commonly account for this risk of bias by stratifying the analysis based on low or high risk of bias in RCTs.

In 2008, the Cochrane Collaboration published a tool and guidelines for the assessment of risk of bias in RCTs [5, 6]. The risk of bias tool was widely embraced by the systematic review community [7]. The tool addresses six domains of bias, classified as low, high, or unclear risk of bias. Domains of bias were selected based on empirical evidence and theoretical considerations that focused on methodological issues likely to influence the results of RCTs.

Several studies reported that the reliability of the risk of bias tool is low [8–10]. Reliability of the risk of bias tool can be assessed between two raters of the same research group when, for instance, they assess the risk of bias of RCTs included in a meta-analysis in duplicate. It can also be assessed across research groups if the risk of bias was assessed for a trial included in two different meta-analyses by two different research groups. Disagreements between two raters of the same research group may be less problematic since they will normally discuss their ratings to come to a consensus. Disagreements between raters from different research groups will be more problematic, for example, if for the same outcome a trial is considered at low risk of bias in one meta-analysis, but is at high risk of bias in another one. Low reliability of risk of bias assessments can then ultimately have repercussions on decision-making and quality of patient care [11, 12].

We recently found that reliability of the risk of bias tool might improve if raters receive intensive standardized training [8]. However, to our knowledge, no formal evaluation of such a training intervention has been performed. We therefore aimed to investigate whether training of raters, with objective and standardized instructions on how to assess risk of bias, would improve the within and between pairs of rater reliability of the Cochrane risk of bias tool.

## Methods

### Study design

In this prospective pilot study, we randomly allocated inexperienced raters to two different levels of training on risk of bias assessment, minimal training or intensive standardized training, for the assessment of the reliability of the risk of bias tool under these different training conditions. The objective of the study was to determine whether standardized intensive training might be effective in increasing the reliability of risk of bias assessments of inexperienced raters, and if effective, how large the magnitude of the effects on reliability could be. We published a protocol before we began the study [13].

### Literature search and trial selection

To identify RCTs to be used for risk of bias assessment, we searched PubMed from inception to March 20_,_ 2014. The search strategy was published with the study protocol [13]. We included every randomized or quasi-randomized clinical trial in patients with knee osteoarthritis that compared a physical therapy intervention to another physical therapy intervention, sham intervention, or no treatment, and which assessed patient-reported pain. The following physical therapy interventions were considered: land-based exercise, aquatic exercise, manual therapy, electric stimulation therapy, and diathermy. We only considered trials published in English. No further restrictions were applied. Two reviewers independently screened reports for eligibility. Disagreements were resolved by a senior author (BdC).

### Data extraction

We used a standardized, piloted data extraction form to extract information on publication year, sample size, type of intervention, and risk of bias. We assessed risk of bias for selected items of the risk of bias tool, namely sequence of generation, allocation concealment, blinding (participants, personnel (therapist), and assessors), and incomplete outcome data. Although a potentially important source of bias, we did not assess selective outcome reporting in our study because we would not have access to protocols of most or all trials for a proper assessment [7]. Within pairs of raters, data extraction was conducted independently. Disagreements within pairs of raters were solved by discussion within pairs until consensus was reached.

### Training on risk of bias assessment

A detailed description of the training method was previously published [13]. Six raters assessed the risk of bias of every included trial. Four of these raters were doctoral students of physical therapy without previous experience in risk of bias assessment, and two raters were experienced risk of bias assessors, who served as reference. We used computer-generated simple randomization to allocate two inexperienced raters to minimal training and two to intensive standardized training. Randomization was performed remotely by one of the authors (SAO) who had no contact with the students. Students were not informed to which training group they were randomized, and they were instructed not to discuss their training with each other to minimize the risk of contamination [5]. After the data extraction was completed, we asked students to guess in which group they were allocated, whether there were any events during data extraction that made them aware of their group allocation, and if this affected their performance in this study.

#### Standardized, intensive training

Inexperienced raters allocated to this group received a 60-min lecture on the definition and importance of each of the assessed domains of bias. In addition, they received standardized instructions on how to assess each of the domains. A detailed description of the training methods is provided in the published protocol [13]. The standardized instructions were based on the Cochrane Handbook [5] and adapted as deemed necessary to increase their objectivity and thus minimize misinterpretations for the assessment of trials of physical therapy in patients with knee osteoarthritis. One of the experienced raters (BdC) discussed these instructions with them, and the students then assessed the risk of bias in a purposively selected sample of ten articles, which were not part of the final study sample. One of the experienced raters (BdC) discussed their assessments after five and ten training articles were assessed. The assessments of the inexperienced raters allocated to intensive standardized training were thus calibrated with the assessments of the experienced rater. The entire training duration was approximately 8 h.

#### Minimal training

Inexperienced raters allocated to this group attended the same 60-min lecture on the definition and importance of each of the assessed domains of bias, without specific or standardized instructions on how to conduct the assessment. In addition, the inexperienced raters were provided with an article, as an optional reading material, which described the risk of bias tool [6] as well as chapter 8 of the Cochrane Handbook for Systematic Reviews of Interventions [5], which specifically addresses the assessment of risk of bias of trials included in a systematic review.

#### Reference assessments

Two experienced raters assessed the risk of bias in all trials using the same standardized instructions used by the intensive standardized training. The risk of bias assessment, after consensus was reached between the experienced raters, was considered the reference assessment in the present study.

The students in both groups were instructed not to discuss their risk of bias assessment with others. The study protocol was approved by the research ethics committee of the Florida International University (IRB-14-0110). We obtained written informed consent from each student.

### Analysis

The primary outcome of our study was between-group reliability, defined as the agreement of the risk of bias assessments of inexperienced raters with the reference assessments of experienced raters. Consensus-based assessments were used for this purpose. The secondary outcome was within-group reliability, defined as the agreement of assessments within pairs of inexperienced raters. We calculated the chance-corrected weighted Kappa with 95% confidence intervals to quantify agreement within and between groups of raters for each of the domains of the risk of bias tool. Assessments falling in the main diagonal (complete agreement) received a weight of 1, assessments adjacent to the main diagonal received a weight of 0.8, and all other assessments received a weight of 0. Weighted Kappa values between 0.93 and 1.00 represent excellent agreement; 0.81–0.92 very good agreement; 0.61–0.80 good agreement; 0.41–0.60 fair agreement; 0.21–0.40 slight agreement; 0.01–0.20 poor agreement; and 0.00 or less no agreement [14]. To compare groups, we calculated the differences in within and between groups of raters Kappa values. We bootstrapped the difference in Kappa values using bias correction and acceleration to derive 95% confidence intervals and *p* values [15]. Assumptions used for the power analysis are presented elsewhere [13].

To explore whether quality of reporting influences agreement, we stratified the analysis according to publication date (before the latest Consolidated Standards of Reporting Trials (CONSORT) statement revision in 2010 [16] vs 2010 and later), assuming that reporting quality of RCTs in physical therapy improved after the publication of the CONSORT 2010 statement [17, 18]. To investigate whether sample size influences agreement, we stratified the analysis by trial size (<50 and ≥50 patients randomized per trial arm), assuming that trial size is associated with methodological quality [19]. All *p* values are two-sided. Analyses were conducted in Stata, release 14 (StataCorp, College Station, TX, USA).

## Results

**Fig. 1:**
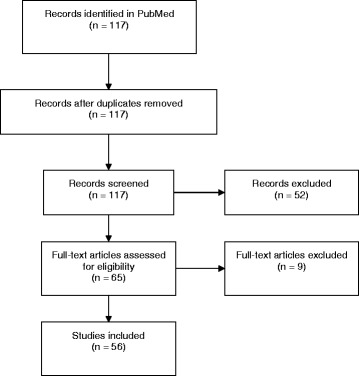
Flow diagram displaying results of literature search

Figure 1 displays the results of our literature search. Our search identified 117 references for screening, and 56 trials including a total of 5182 patients were included in our analysis. The median year of publication was 2009, ranging from 1995 to 2013, and the median number of randomized patients was 65, ranging from 20 to 439. Table 1 displays the risk of bias in included trials. Based on the consensus of experienced raters, most trials had a high risk of performance bias due to inappropriate or lack of blinding of patients (59%) or blinding of therapists (91%). Reporting of methods used to conduct randomization was often problematic, with unclear risk of bias for random sequence of generation in 27 trials (48%) and for allocation concealment in 42 trials (75%).

**Table 1:** Risk of bias of trials included in the present study^a^

Source of bias	Low risk	Unclear risk	High risk
Random sequence generation	29 (52)	27 (48)	0 (0)
Allocation concealment	13 (23)	42 (75)	1 (2)
Blinding of patients	16 (29)	7 (13)	33 (59)
Blinding of therapists	4 (7)	1 (2)	51 (91)
Blinding of outcome assessors	22 (39)	12 (21)	22 (39)
Incomplete outcome data	17 (30)	13 (23)	26 (46)

Displayed values are number of trials and percentage

^a^From consensus between a pair of experienced raters

### Between-group reliability: agreement of inexperienced raters with reference

**Fig. 2:**
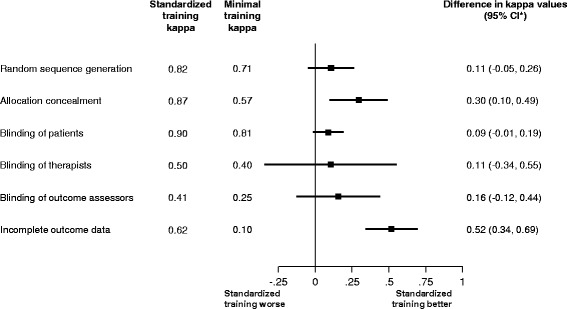
Difference in the agreement of inexperienced raters with reference. Difference in agreement of assessment between minimal training raters and experienced raters and between standardized training raters and experienced raters. *Bootstrapped 95% confidence intervals

Figure 2 displays the Kappa values for agreement between intensive and minimal training groups and the reference of experienced raters, as well as the difference in these Kappa values between groups. Kappa values between the standardized training group and reference ranged from 0.41 (fair agreement) for blinding of outcome assessors to 0.90 (very good agreement) for blinding of patients. Kappa values between the minimal training group and reference across items of the risk of bias tool ranged from 0.10 (poor agreement) for incomplete outcome data to 0.81 (very good agreement) for blinding of patients. Kappa between the standardized training group and reference was higher than the agreement between the minimal training group and reference for all risk of bias items. The difference in Kappa values ranged from 0.11 to 0.52. The difference reached conventional levels of statistical significance for allocation concealment (difference in Kappa 0.30, *p* = 0.004) and incomplete outcome data (difference in Kappa 0.52, *p* < 0.001). There was no evidence of an interaction between the difference in Kappa values and trial size for all risk of bias items (*p* ≥ 0.10, Additional file 1: Figure S1). There was evidence for an interaction between differences in Kappa values and period of publication before or after publication of the CONSORT 2010 statement, with difference in Kappa values more pronounced after publication of the CONSORT 2010, for the assessment of incomplete outcome data (*p* = 0.002), but not for any other risk of bias items (*p* ≥ 0.07, Additional file 2: Figure S2).

### Within-group reliability: agreement within pairs of inexperienced raters

**Fig. 3:**
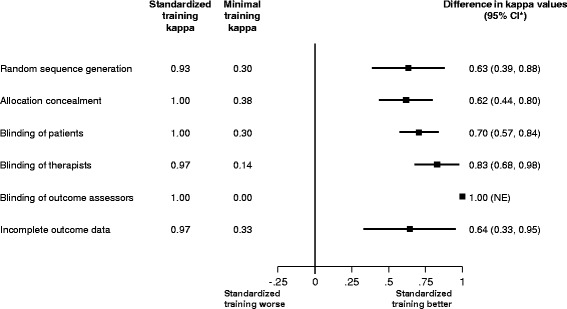
Difference in agreement within pairs of inexperienced raters. *Bootstrapped 95% confidence intervals. *NE* not estimable

Figure 3 displays the Kappa values for agreement within pairs of inexperienced raters, for the minimal and standardized training groups, and the difference in Kappa values between groups. Kappa values in the standardized training group ranged from 0.93 (excellent agreement) for random sequence of generation to 1.00 (perfect agreement) for allocation concealment, blinding of patients, and blinding of outcome assessors. Kappa values in the minimal training group ranged from 0.00 (no agreement) for blinding of outcome assessor to 0.38 (slight agreement) for allocation concealment. There was strong evidence (*p* < 0.001) indicating that, for all risk of bias items, within-group agreement in the standardized training group was higher than in the minimal training group. The difference in Kappa values ranged from 0.62 for allocation concealment to 1.00 for blinding of outcome assessors.

## Discussion

To our knowledge, this prospective pilot study is the first to indicate that the reliability of the risk of bias tool may be improved by a standardized training of inexperienced raters. Increase in between-group Kappa agreement ranged from 0.09 to 0.52 across risk of bias items, but only reached statistical significance for allocation concealment and incomplete outcome data. These results indicate that intensive standardized training may minimize the variation in risk of bias assessment across different research groups. Increase in within-group Kappa agreement ranged from 0.62 to 1 across risk of bias items, and there is strong evidence that standardized training will improve within-group Kappa agreement for all risk of bias items.

Critics of the risk of bias tool commonly refer to its low agreement within a pair of raters to challenge its usefulness [8, 10, 20, 21]. Indeed, Kappa within a pair of raters for the Cochrane risk of bias tool has been reported to be generally low [8, 9, 22]. Our findings are in line with previous studies, in that we also observed a rather low agreement within a pair of inexperienced raters that received minimal training, with Kappa values indicating only a slight agreement at best. However, the practical implications of such disagreement may be irrelevant, since raters within a research group usually discuss to reach consensus when their assessments differ. What is more relevant is whether or not their risk of bias assessment after discussion is accurate, and whether assessments are similar to those from other research groups, since low agreement of risk of bias assessments between research groups can have repercussions on decision-making and quality of patient care [11, 12]. Our results suggest that, although a discussion between minimal training raters to reach consensus will lead to a more accurate risk of bias assessment, it will not reach an acceptable level of agreement between different research groups. These findings are in agreement with Hartling et al. and Armijo-Olivo et al., who investigated the agreement between pairs of raters from different research groups, and also concluded that discussion within pairs of raters to reach consensus is not enough to reach acceptable levels of agreement across different research groups [8, 10].

Although low agreement of the Cochrane risk of bias tool has been reported by several studies, none have proposed and investigated ways to improve it. Our study is the first to show that an intensive standardized training on risk of bias shows promise as a method to improve agreement not only within pairs of raters, but also across research groups. We found that standardized training improves agreement of all items assessed within a pair of raters. Although standardized training also led to better agreement between pairs of raters for all items assessed, there was only evidence of improvement for concealment of allocation and incomplete outcome data risk of bias assessment. In the present study, assessment of concealment of allocation was most problematic, with 75% of the trials not reporting enough information to allow a proper assessment of this item. Raters receiving standardized training, including explanations and decision rules, had higher agreement between pairs of raters, notwithstanding poor reporting of the item. As a way to circumvent poor reporting of randomization methods, Corbett et al. suggested that reviewers take between-group baseline imbalances in important prognostic indicators into consideration when assessing selection bias, something that could also be included in standardized instructions to further facilitate the risk of bias assessment of this poorly reported item [23]. The largest difference in agreement between pairs of raters receiving standardized training was observed for the assessment of incomplete outcome data. Savović et al. conducted a survey with stakeholders within the Cochrane Collaboration and reported that most of them (67%) found the assessment of risk of bias due to incomplete outcome data to be the most difficult [24]. Such difficulty may explain the largest improvement observed in the agreement between pairs of raters with standardized training where clear instructions were provided on how to assess this item.

The standardized instructions and training for risk of bias assessment should be tailored to address the main methodological problems commonly found in the area of research of interest. For instance, for most physical therapy interventions, it is difficult if not impossible to blind the therapist. However, a trial comparing two different spinal manipulation techniques will not necessarily have a high risk of performance bias due to the lack of therapist blinding. This problem can be circumvented, for example, by using expertise-based randomization, where patients are only treated by experts on a particular intervention [25]. In order to develop valid instructions for risk of bias assessment within a specific area of research, it is of utmost importance that experienced epidemiologists in this area of research are involved in the process so that risk of biases and possible ways to minimize them are properly identified and addressed in the instructions. Properly developed instructions for risk of bias assessment will not only improve the agreement of the risk of bias tool within- and between-research groups, but will likely also increase the validity and transparency of the risk of bias assessment process within a specific area of research.

The main strength of our study is that we included raters completely inexperienced with the risk of bias assessment to investigate the effect of standardized training on the agreement of the risk of bias tool. The randomization of only inexperienced raters to training groups allowed us to maximize the effect of standardized training. If raters were already experienced with the risk of bias assessment, there could be limited room for improvement as postulated in a previous study that investigated the effect of training on a similar method for methodological quality assessment [26]. The main limitation of the present study was the low number of raters randomized to training groups. While this was unproblematic for statistical precision, we cannot exclude relevant baseline imbalances that could partially explain the observed results. To try and overcome this limitation, an obvious strategy would be to assess the baseline agreement between risk of bias assessment from each inexperienced rater with those from experienced raters and then match inexperienced raters in accordance to their baseline performance to conduct a matched-pairs randomization. However, baseline assessment of students’ performance in risk of bias assessment could already result in training, which in turn could bias the results of our analysis.

Our results could potentially be influenced by performance bias resulting from a nocebo effect in the control group of doctoral students who received minimal training. If students in the control group understood that they were not receiving the best training available in our study, they could have felt discouraged to try and perform risk of bias assessments to the best of their ability. This could in turn lead to an artificially lower agreement of the risk of bias tool with minimal training as compared to standardized training. Unblinding could also have resulted in an underestimation of the difference in between-group reliability across groups of raters, since inexperienced raters in the minimal training group could alternatively have sought additional training elsewhere or be prompted to self-study. To try and minimize the risk of such performance bias, inexperienced raters were not informed to which training group they were randomized, and they were instructed to not discuss with each other any characteristics of their training. After data extraction was completed, inexperienced raters were asked to guess their group assignment. All four inexperienced raters correctly identified the groups to which they were allocated, but reported that their suspicion did not influence their performance. Moreover, the use of minimal training as a control intervention may have led to an underestimation of the effect of our standardized training. Although “no training” could be used as a control intervention instead of minimal training to maximize the effect of standardized training, this could have substantially increased the risk of performance bias in our study as explained above. Finally, the minimal training used in the present study may be better than what reviewers commonly receive. Again, the effect of intensive training may be even larger in a setting where minimal training is worse than the minimal training provided here.

The low number of raters randomized to intervention groups limits the generalizability of our findings and may have generated confounding as previously mentioned. Because it is a pilot study, we included the minimal number of participants needed to calculate Kappa agreements within each study condition. Given the promising large effect of standardized training observed in the present study, a future study using the same methods but including a larger number of inexperienced raters should be conducted. Generalizability may be further limited by the characteristics of the trials assessed in our study. Reliability of the risk of bias assessment could vary if trials with different patient populations, interventions, and outcomes were assessed. However, we believe the sample of trials used allowed us to make a more valid assessment of blinding, given the subjective nature of pain outcomes, and the difficulties involved in blinding of patients and therapists in physical therapy trials. Our results are further limited by the exclusion of selective reporting of outcomes assessment from our investigation.

## Conclusions

Intensive and standardized training on risk of bias assessment significantly improved the within-group agreement for all items assessed. There is also evidence that it may lead to a significant improvement in the between-group agreement of allocation concealment and incomplete outcome data assessment. There is some indication that it may also improve the between-group agreement for the remaining items, but given the already good to excellent agreement in the absence of standardized training for some of the items assessed, the net gain for these items may be limited. Nonetheless, we provide evidence that the reliability of the Cochrane risk of bias tool may be generally improved with the implementation of an intensive, standardized training.

## Additional files

Additional file 1: Figure S1. Difference in the agreement of inexperienced raters with reference stratified by trial size. Agreement assessment between minimal training raters and experienced raters and between standardized training raters and experienced raters. *Bootstrapped 95% confidence intervals. (PPTX 49 kb)
Additional file 2: Figure S2. Difference in the agreement of inexperienced raters with reference stratified by year of publication before or after the CONSORT 2010. Agreement assessment between minimal training raters and experienced raters and between standardized training raters and experienced raters. *Bootstrapped 95% confidence intervals. (PPTX 49 kb)

## Abbreviations

CONSORT: Consolidated Standards of Reporting Trials
RCT: Randomized clinical trial
