# Supplementary figures and images for: Effect of standardized training on the reliability of the Cochrane risk of bias assessment tool: a prospective study
Source: Syst Rev. 2017 Mar 3;6:44. doi: 10.1186/s13643-017-0441-7 (PMC5335785; doi:10.1186/s13643-017-0441-7)

## Slide 1
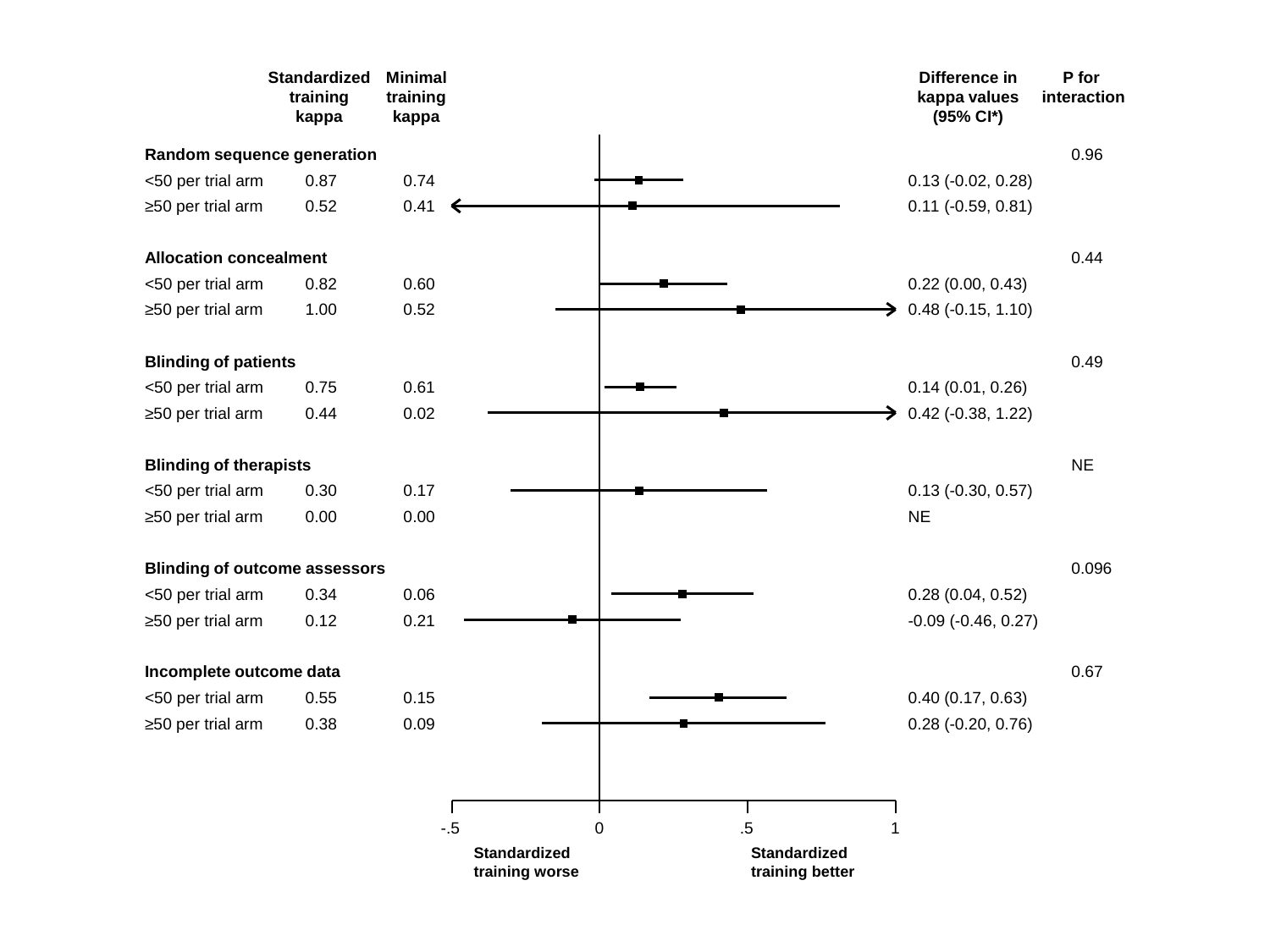

Supplement: Additional file 1: Figure S1. — Difference in the agreement of inexperienced raters with reference stratified by trial size. Agreement assessment between minimal training raters and experienced raters and between standardized training raters and experienced raters. *Bootstrapped 95% confidence intervals. (PPTX 49 kb) [file 13643_2017_441_MOESM1_ESM.pptx]

## Slide 1
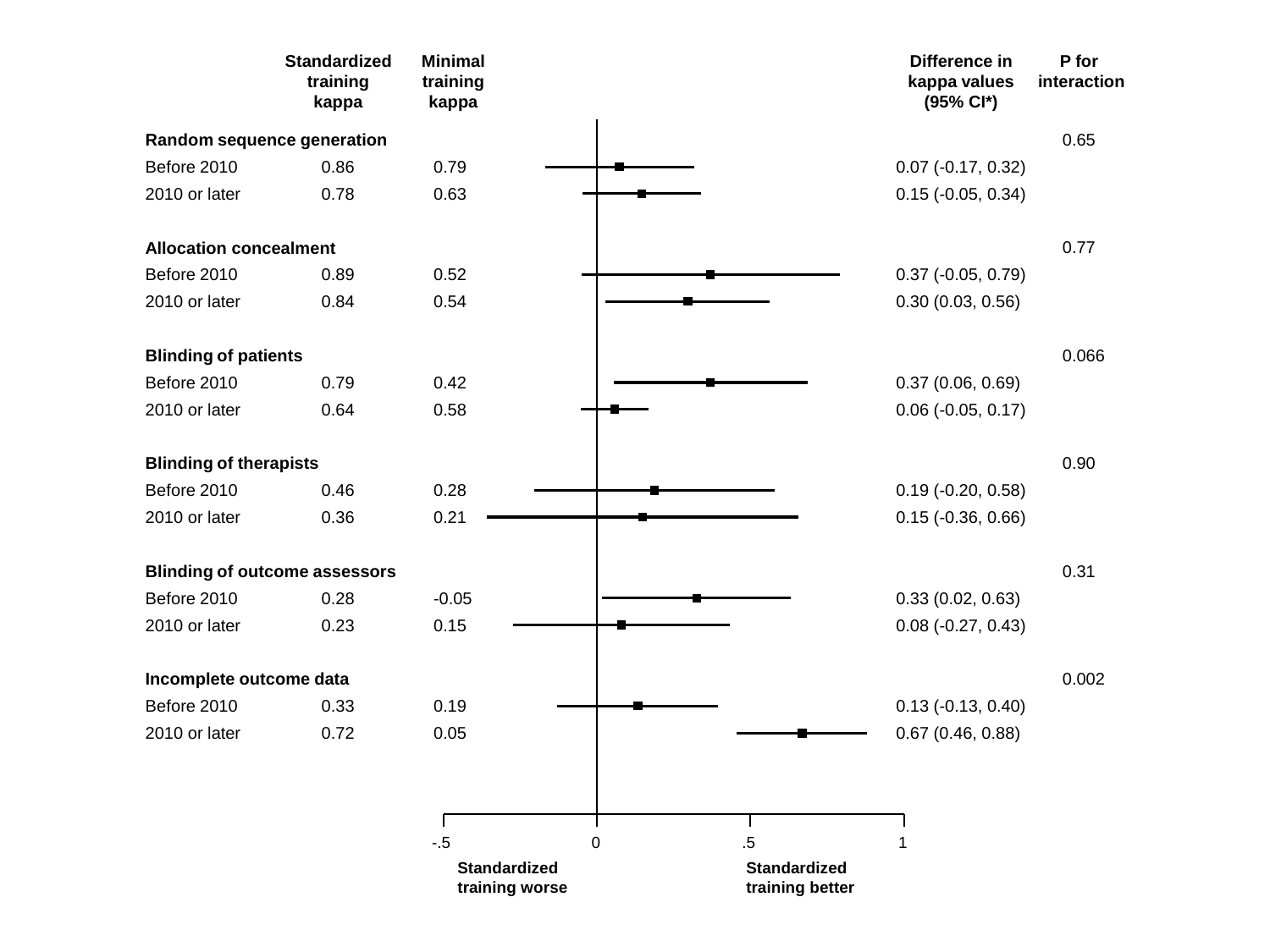

Supplement: Additional file 2: Figure S2. — Difference in the agreement of inexperienced raters with reference stratified by year of publication before or after the CONSORT 2010. Agreement assessment between minimal training raters and experienced raters and between standardized training raters and experienced raters. *Bootstrapped 95% confidence intervals. (PPTX 49 kb) [file 13643_2017_441_MOESM2_ESM.pptx]
